# Sensory receptors—design principles revisited

**DOI:** 10.3389/fncel.2013.00001

**Published:** 2013-01-24

**Authors:** Dieter Wicher

**Affiliations:** Laboratory of Reception and Transduction, Department of Evolutionary Neuroethology, Max Planck Institute for Chemical EcologyJena, Germany

This research topic was aimed toward collecting the present knowledge of structure and function of sensory receptors in animal kingdom as well as the mechanisms of signal transduction and amplification. To translate external signals such as light, sound, smell, etc., into an appropriate intracellular signal, sensory receptors use either a fast, direct or a slow, indirect way. These qualitatively different signal transduction pathways are now usually called ionotopic or metabotropic. Historically, the term metabotropic receptor has been introduced to distinguish a subtype of glutamate receptors that triggers chemical reactions (cell metabolism) in the postsynaptic cell from other glutamate receptors that pass an ion current (ionotropic) (Eccles and McGeer, 1979). Metabotropic glutamate receptors were found to be linked to inositol phospholipid metabolism (Sugiyama et al., 1987), and were subsequently identified as G-protein-coupled receptors (GPCRs) (Masu et al., 1991). The terminology ionotropic/metabotropic has been extended to other neurotransmitter receptors, such as for nicotinic/muscarinergic acetyl choline or GABA_A_/GABA_B_ receptors. All metabotropic neurotransmitter receptors are GPCRs. There are, however, a large number of non-GPCRs that also fulfill the original definition for a metabotropic receptor, namely “that the transmitter acts *indirectly*, by triggering a chemical reaction or a series of reactions” (Eccles and McGeer, 1979). Accordingly, it has been used to extent the term metabotropic receptor to receptor kinases, receptor cyclases, etc., as well.

Sensory receptors are often part of complex signal transduction cascades. An ion current through an ionotropic receptor may initiate metabotropic signaling, as well as a metabotropic receptor may downstream affect the function of ion channels. An example for protein–protein interaction in chemosensation is given in the original article by Liu et al. (2012). The authors identified so far unknown binding partners of Gγ13, a G-protein subunit expressed in mammalian taste and olfactory receptor cells. These binding partners are PDZ-domain containing proteins assumed to target Gγ13 to specific subcellular locations or represent parts of the chemosensory signal transduction cascade.

The evolution of chemoreceptors shows that—from bacteria to mammals—both, ionotropic as well as metabotropic mechanisms were conserved. Functional aspects of chemoreceptors, including the interaction of electrical and chemical signaling, and the amplification of sensory information are discussed in the perspective article (Wicher, 2012). Intriguingly, insect chemoreceptors operate as ionotropic receptors, namely odorant receptors (ORs), ionotropic glutamate-like receptors (IRs), and gustatory receptors (GRs). Getahun et al. (2012) investigate the temporal response dynamics of insect chemoreceptors and demonstrate that olfactory sensory neurons (OSNs) expressing ORs, GRs, or IRs differ in their response kinetics to brief stimuli. OR-expressing neurons respond faster and with higher sensitivity, while IR-expressing neurons do not adapt to long stimulations. Although ORs primarily operate as ionotropic receptors, metabotropic signaling was seen to modulate the ionotropic odor response (Olsson et al., 2011; Sargsyan et al., 2011). Stimulation of cAMP production enhanced the response to a given odor concentration, corresponding to an increased sensitivity. This type of modulation may constitute the mechanistic basis for the higher sensitivity of ORs compared with IRs.

Chemical information released from different sources may interfere during processing in the nervous system and affect the response of an organism. Odor mixtures can act in synergistic or in an inhibitory way. On the level of the chemoreceptors the existence of a huge number of different chemical signal molecules leads to the intriguing question of receptor specificity and whether a given chemical signal is perceived independent of the background. The interaction of odorant and pheromone detection in moths is reported by Pregitzer et al. (2012) and commented by Anton and Renou (2012). Certain plant odors are known to inhibit the activation of pheromone receptors. The reported investigations provide evidence that the odorant-pheromone interaction already takes place at the receptor level.

Since the first editorial to this topic was written in 2010 recent progress shed new light on structure and function of certain receptors. Channelrhodopsins, for example, are photoreceptors in green algae which conduct a current upon illumination. They are seven transmembrane (7-TM)-spanning proteins as typical for GPCRs but do not couple to a heterotrimeric G-protein. With the given 7-TM topology it was as yet not clear how the channelrhodopsin proteins have to arrange to form an ion channel. Recently, the non-selective cation channel, channelrhodopsin-2 from *Chlamydomonas reinhardtii* has been successfully crystallized (Müller et al., 2011; Kato et al., 2012). The channelrhodopsin-2 proteins were found to stably dimerize in such an arrangement that the third and the fourth TM helix of each protein align to a tetramer thereby lining the cation-permeable pore. Another example for ion channel-forming 7-TM proteins are the above mentioned insect ORs. In contrast to homodimeric channelrhodopsin channels they are heterodimers, composed of variable, odorant-binding protein OrX, and an ubiquitous co-receptor OrCo. There is growing evidence that both OR proteins contribute to channel pore formation and determine their properties such as the ion permeability and pharmacological properties (Nichols et al., 2011; Pask et al., 2011; Nakagawa et al., 2012). It remains to be established whether OrCo form homomeric channels in the receptor neurons as seen in the heterologous expression system and whether they represent the metabotropic pathway used to tune the sensitivity of the ionotropic receptor (Olsson et al., 2011; Sargsyan et al., 2011). The role of stimulatory G-proteins in olfactory signaling has been demonstrated (Deng et al., 2011), and also downstream signaling such as cAMP production were seen to affect the odor response of receptor neurons (Olsson et al., 2011). These recent findings on insect OR function modify the view to classify them. While in the first editorial they have been considered as combined metabotropic and ionotropic receptors, they might now be more appropriately characterized as metabotropically regulated ionotropic receptors. This change of view illustrates the highly dynamic development in the field.
